# Cell-associated viral community composition and its functional potential in a dimictic lake on the Canadian shield

**DOI:** 10.3389/fmicb.2026.1941165

**Published:** 2026-10-27

**Authors:** Cody C. Collis, Ellen S. Cameron, Kirsten M. Müller, Monica B. Emelko, Nicolas Tromas, Jozef I. Nissimov

**Affiliations:** 1 Department of Biology, University of Waterloo, Waterloo, ON, Canada; 2 Department of Civil and Environmental Engineering, University of Waterloo, Waterloo, ON, Canada; 3 CARRTEL-INRAE, Thonon-les-Bains, France; 4 Department of Microbiology and Immunology, McGill University, Montréal, QC, Canada

**Keywords:** auxiliary metabolic genes, bacteriophages, *Caudoviricetes*, diversity, lake, metagenomics, microbial community

## Abstract

The Turkey Lake Watershed (TLW), located in Northern Ontario, forms a cascading lake system that drains into Lake Superior. Historically, the TLW has served as a key research site for studies on acid rain and climate change. Despite its long-standing ecological importance, investigations into its microbial communities remain limited, and to date, no studies have explicitly examined the viral component of this watershed. Viruses have been frequently implicated in nutrient cycling in aquatic environments and strongly influence their microbial hosts. Therefore, viral ecology offers a powerful way to extend the environmental legacy of the TLW by identifying the biological mechanisms through which long-term changes in acid deposition, climate, hydrology, carbon chemistry, and forest disturbance propagate through microbial food webs. First steps can be a preliminary view into the type of viruses that are present, and information on which microbial hosts they may be infecting. We conducted a seasonal metagenomic survey of Big Turkey Lake (BTL)—one of the primary lakes within the TLW—from summer 2018 to winter 2020. Our study characterized the diversity and composition of cell-associated viral communities, predicted potential host relationships, and identified auxiliary metabolic genes (AMGs) to better understand the functional roles of these viruses. The results revealed a dynamic viral community that varied across seasons. Most viral contigs were classified as *Caudoviricetes*, while members of *Phycodnaviridae* emerged as important non-bacteriophage contributors to the viral assemblage. We reconstructed six draft viral metagenome-assembled genomes (vMAGs), ranging in size from 11 to 45 kbp, all identified as *Caudoviricetes,* with two being potential temperate phages and a third containing ribosomal genes. Notably, AMGs associated with lipid metabolism were detected exclusively in winter, suggesting a potential season- or niche-specific role for these genes in BTL. AMGs related to phosphorus acquisition were present in all samples, suggesting a potential coupling between transport dynamics of dissolved organic matter and phosphorus previously described in the TLW, and the host-virus dynamics described here. Together, these findings provide the first insight into the viral ecology of the TLW and establish a foundation for future studies investigating the roles of viruses in shaping the microbial dynamics in this important habitat.

## 1 Introduction

Viruses are integral components of microbial communities in aquatic environments, where they play crucial roles in biogeochemical cycling, regulation of primary production, and bacterial mortality (Suttle, 2007; Mojica et al., 2016; Mojica and Brussaard, 2014). By infecting and lysing host cells, viruses influence nutrient turnover and energy flow. They also shape bacterial and microalgal activity through the expression of auxiliary metabolic genes (AMGs)—genes acquired via evolutionary processes such as horizontal gene transfer during infection (Breitbart et al., 2007). Although these genes typically do not play a direct role in virus replication, they can enhance host metabolism, affecting key cellular functions. In aquatic systems, AMGs are implicated in processes such as photosynthesis, carbon metabolism, cofactor biosynthesis, and nutrient cycling (Hurwitz and U’Ren, 2016; Crummett et al., 2016; Meza-Padilla et al., 2024). Notably, approximately 40% of *psbA* (photosystem II) expression in marine systems is virus-derived (Sieradzki et al., 2019), underscoring the ecological significance of viruses and their AMGs.

While the majority of research on aquatic viruses has focused on marine ecosystems, recent advances have revealed a remarkable diversity of viral and microbial communities in freshwater environments (Thompson et al., 2017; Zhou et al., 2025). Seasonal patterns in viral abundance and community composition are largely governed by host availability, which is influenced by bottom-up factors such as nutrient supply and top-down factors such as predation (Zhou et al., 2025). In typical freshwater lakes, seasonal phytoplankton succession—for example, shifts between diatoms and cyanobacteria driven by changes in temperature, light, and nutrient conditions—can strongly influence the composition of viral populations (Wentzky et al., 2020; Wei et al., 2020).

Despite these advances, major gaps persist in our understanding of viral infection dynamics and metabolic capabilities in most aquatic environments (Sime-Ngando, 2014; Bonetti et al., 2019; Pradeep Ram and Sime-Ngando, 2023). One area of uncertainty is the role of viruses during harmful algal blooms (HABs). Some studies suggest that viruses contribute to bloom termination (Grasso et al., 2022), while others indicate that viral infections can exacerbate HAB effects—viral lysis may release intracellular toxins into the water column, facilitating their persistence in dissolved form (Steffen et al., 2017; McKindles et al., 2020; Lee et al., 2023). The function of AMGs during HAB events also remains poorly understood. For instance, a cyanophage AMG involved in host phycobilisome degradation may help sustain infection under high light and nutrient-limited conditions (Meza-Padilla et al., 2024), but the prevalence of this and similar genes in freshwater viral communities is still unclear.

Differences in scale and physical structure mean that findings from marine studies cannot always be extrapolated to lakes. Freshwater systems are generally shallower and enclosed, making them more sensitive to local factors such as eutrophication and climate change (Adrian et al., 2009; Williamson et al., 2008). Understanding viral roles in these ecosystems is thus essential for predicting how microbial communities respond to environmental stressors.

A crucial first step toward this understanding is characterizing the composition and relative abundance of viruses in freshwater environments, including those associated with host cells. However, sampling viruses in these settings poses practical challenges, particularly in remote habitats such as inland lakes of Northern Ontario. Unlike oceanic expeditions where onboard facilities allow collection of large water volumes, inland lake sampling is often limited by equipment and accessibility, making traditional virus concentration methods such as TFF of large volumes (Wommack et al., 2010) or chemical flocculation (John et al., 2011), impractical. An alternative is to focus on the cell-associated viral fraction, which includes viruses attached to or potentially infecting host cells. This approach simplifies field collection, requiring only filtration of host cells rather than extensive water concentration procedures.

Here, we used shotgun metagenomics to characterize seasonal variation in the relative abundance of cell-associated viruses in Big Turkey Lake, a dimictic lake which is part of the Turkey Lake Watershed (TLW) in Northern Ontario, Canada. We also investigated potential viral hosts and identified AMGs that may mediate host metabolic processes. To our knowledge, this represents the first study of viral communities in the TLW, establishing a foundation for future research into the ecological roles and functional potential of freshwater viruses in this watershed.

## 2 Materials and methods

### 2.1 Study site and sampling

This study was conducted using samples obtained between 2018 and 2020 (Supplementary Table S1) from Big Turkey Lake (BTL; 47 02′ 54.7” North, 84 25′ 19.3” West) within the Turkey Lake Watershed (TLW; Figure 1A), located approximately 50 km north of Sault Ste. Marie, Ontario. The watershed, which consists of several holomictic lakes (i.e., lakes which experience complete water column mixing in spring and autumn), represents a relatively undisturbed habitat by human activity, and has been the prior focus of studies on the effects of acid rain and other aspects related to climate change (Jeffries and Foster, 2001). BTL is the largest lake within the TLW and is classified as dimictic (e.g., its water fully mixes top-to-bottom usually twice yearly) and oligotrophic (Jeffries et al., 1988). Biomass samples were collected in July 2018, monthly over a period from May 2019–August 2019, and in January 2020. Briefly, 1 L of water (Supplementary Table S1) was collected in triplicates at Secchi depth and vacuum filtered through 47 mm Whatman GF/C filters, then kept at −20 °C until downstream processing (Cameron, 2021). For samples collected during a period of ice cover (e.g., January 2020), samples were taken from surface water (e.g., ~25 cm below the ice).

**Figure 1:**
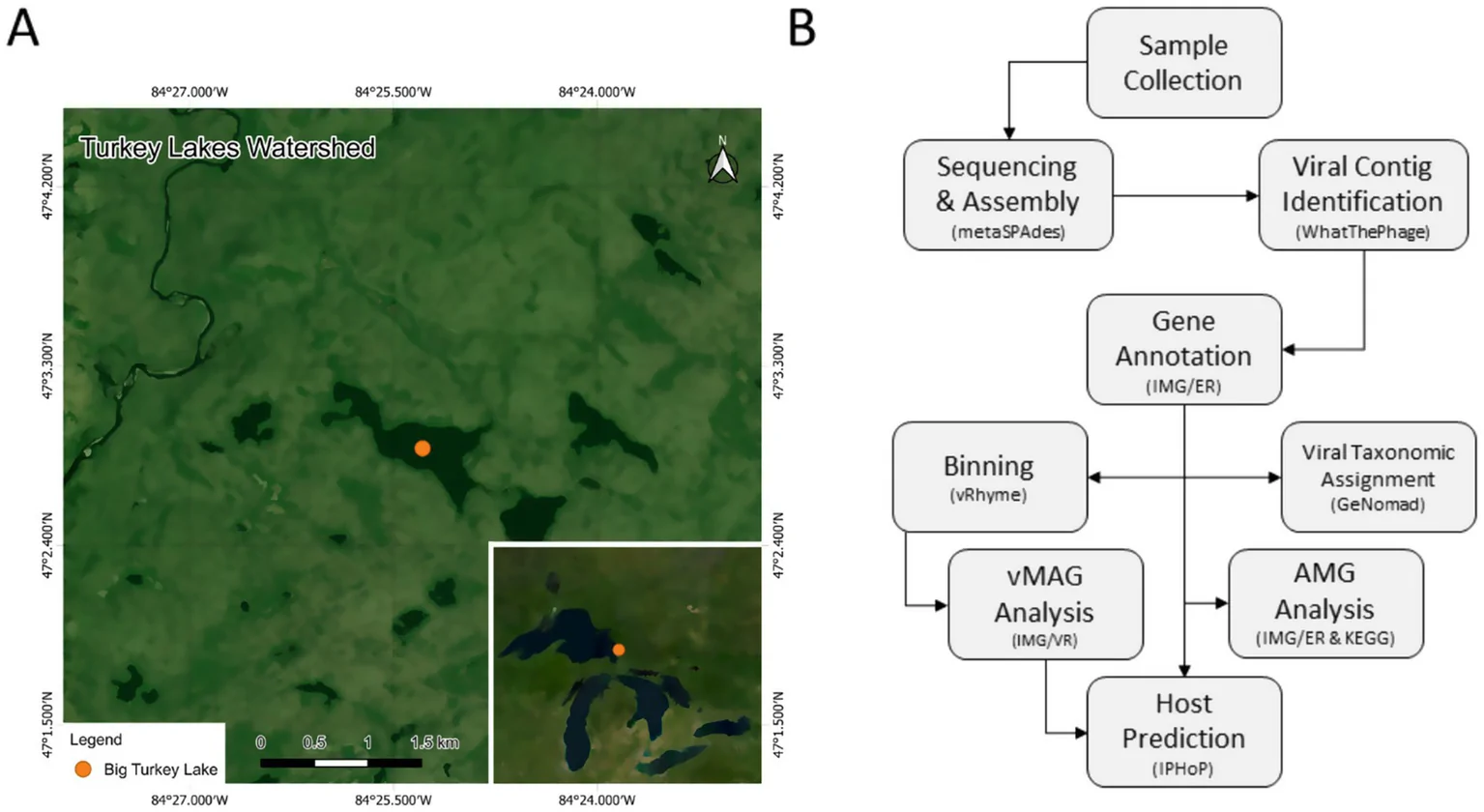
The sampling location of this study was Big Turkey Lake (BTL), within the Turkey Lake Watershed (TLW), which is adjacent to Lake Superior, Canada **(A)**. The study consists of biomass samples collected during July 2018 – January 2020 for shotgun metagenomics. Extracted nucleic acids were subject to sequencing and a bioinformatics workflow **(B)** for assessing the microbial cell-associated viral communities.

### 2.2 Sequencing, assembly, and analysis

Although we collected biological replicates, only a single replicate from each time point was sent for sequencing in our study (the other replicates were used for a different study). For the sample taken in July 2018, DNA extraction was performed using the Qiagen DNeasy PowerSoil kit, with shotgun sequencing being done by Metagenom Bio. Inc. (Waterloo, Ontario) with Illumina MiSeq 250×2 paired-end sequencing. For samples taken in 2019 and 2020, DNA extraction and shotgun sequencing was performed by the University of Toronto’s Centre for the Analysis Genome Evolution and Function using the Qiagen DNeasy PowerSoil kit, and Illumina MiSeq 250×2 paired-end sequencing, respectively. Raw reads (Supplementary Table S2) were subsequently put through the Metagenome-atlas pipeline (ver. 2.9.0) with default parameters for both quality control and assembly; specifically, the pipeline uses the BBtools suite for trimming and filtering reads, and we used MetaSPAdes with default parameters as the assembler (Kieser et al., 2020; Bushnell, 2014; Nurk et al., 2017). Bioinformatics analysis was as described below and in Figure 1B.

### 2.3 Virus identification and taxonomic classification

To identify the viral contigs, a combination of VIBRANT and VirSorter2 was used (Kieft et al., 2020; Guo et al., 2021) from within the WhatThePhage pipeline (ver. 1.1.0) (Marquet et al., 2022), with a minimum cutoff of 1,500 bp and default parameters chosen for both identifiers. Contigs identified as viral by at least one identifier were retained. Gene annotation for viral contigs was performed using JGI’s Integrated Microbial Genomes with Microbiome Samples – Expert Review (IMG/ER) resources (Chen et al., 2021). Taxonomic evaluation of the viral contigs was performed using the *annotate* function of geNomad (ver. 1.11.1) (Camargo et al., 2024) using default parameters, while relative abundance was calculated based on the number of viral contigs in each sample. CheckV was used to determine the validity of the proportion of short (1.5 k-3 kb) viral contigs that geNomad could not classify (ver. 0.9.0) (Nayfach et al., 2021a,b); those with host contamination or with no viral genes were discarded upon verification. Viral contigs were then binned using vRhyme (ver. 1.1.0) (Kieft et al., 2022), with the resulting viral metagenome-assembled genomes (vMAGs) assessed for completeness and contamination using CheckV Those vMAGs that had a completeness score of >90% from CheckV were considered as high-quality MAGs (Nayfach et al., 2021a,b) and used for further analysis. Coverage of the viral contigs was performed using CoverM (ver. 0.6.1) with the contig function, using the --*rpkm, --min-covered-fraction 30,* and *--proper-pairs-only* options (Aroney et al., 2025), and subsequent rarefaction analysis (Supplementary Figure S1) was performed using the *ggrarecurve* function from the MicrobiotaProcess R package (ver.1.15.0) (Xu et al., 2023). For vMAGs that were scored as ‘complete’ by CheckV, visualizations were created using *gbdraw* (Kawato, 2026).

### 2.4 Identification of auxiliary metabolic genes (AMGs) and host predictions

To determine AMGs found within the BTL cell-associated virus community, we followed a similar protocol to the method used by VIBRANT to annotate AMGs (Kieft et al., 2020). In short, all IMG/ER annotations from viral contigs were compared to the KEGG Orthology (KO) database to determine the specific functional potential and pathway of each gene (Kanehisa et al., 2016). Viral annotations were first flagged as candidate AMGs if they were (1) within a viral contig, and (2) were not directly involved with viral replication, DNA synthesis or transcription. Then we examined the flanking regions of the remaining candidates to determine the presence of viral hallmark genes. Specifically, we examined the flanking regions for either known viral genes (e.g., phage terminases), potential viral genes, and regions of short, hypothetical coding sequences that are used by some programs to identify viral contigs (Khot et al., 2020). For the purpose of our work, candidates that had a virus hallmark gene on only one flanking region were deemed as potential AMGs, whereas those for which we identified viral genes on both flanking regions, we deemed as high confidence AMGs. Genes that did not have viral flanking regions were not considered. Host prediction for all viral contigs and constructed vMAGs was performed using the iPHoP pipeline using the default host database (ver. 1.3.1) (Roux et al., 2023), which has been designed to predict bacterial and archaeal hosts of phages based on their genomic sequences within viral contigs.

## 3 Results and discussion

### 3.1 Cell-associated viral composition and abundance in Big Turkey Lake

In our study, the focus was on viruses that were associated with cells (i.e., viruses that were either potentially actively infecting host cells or were attached to their exterior), which resulted in a total of 2.8–4.54 M raw reads (Supplementary Table S2) with sufficient coverage for analysis (Supplementary Figure S1). Note that in our analysis we excluded the July 2019 sample as it resulted in very low coverage and sequencing depth, and hence did not pass our quality control checks. Across the rest of the samples, approximately 81% of total viral contigs were able to be taxonomically classified through geNomad analysis. The remaining viral contigs (approx. 19%) were unable to be classified and represent potentially novel viral sequences.

Within the identified viral contigs in BTL, there were two major classes of viruses represented (Figure 2A): *Caudoviricetes,* otherwise known as bacteriophages, and *Megaviricetes*, large double-stranded DNA viruses that are part of the nucleocytoplasmic large DNA viruses (NCLDVs), capable of infecting a diverse range of eukaryotic organisms (Koonin and Yutin, 2010). In BTL, *Caudoviricetes* displayed the highest relative abundance (RA) of all viruses (71.8% of total viral contigs) and were present in all samples. *Megaviricetes* were also present in all samples, but with a comparatively lower RA (9.1% of total viral contigs). This is in accordance with other studies that have found that bacteriophages are generally more abundant than their NCLDV counterparts in lake environments. For example, in a study of Lake Ontario and Lake Erie, viruses belonging to the previously assigned *Myoviridae* family of bacteriophages comprised ~80% of the viral sequences detected, whereas phycodnaviruses, members of *Megaviricetes*, made up a much smaller portion of the viral community (Mohiuddin and Schellhorn, 2015). Nevertheless, *Megaviricetes*, while less abundant overall, play a significant role in modulating phytoplankton dynamics and influence community structure and succession in most aquatic environments (Gimenes et al., 2012). Importantly, the abundance and diversity of bacteriophages and *Megaviricetes* can be influenced by factors such as the trophic state of a lake, its depth, and seasonal variations such as temperature. In our study, the RA of identified *Megaviricetes* was highest during the summer months of June and August of 2019 (27.7 and 22.6%, respectively). The reverse was seen in May 2019 and January 2020, where the RA of identified *Megaviricetes* were 2.0 and 2.1%, respectively. *Caudoviricetes* display the reverse trends in their RA, with summer abundances being reduced to 35.5% in August 2019 and increasing to approximately 85% in both May 2019 and January 2020. The summer months at BTL typically have temperatures higher than 20 °C whereas winter is typically below freezing with ice cover (Supplementary Table S1). Hence, given that microbial host abundance and composition are known to be impacted by temperature in most aquatic systems, we conclude that temperature naturally also plays a central role in structuring the cell-associated viral abundance and its composition in BTL.

**Figure 2:**
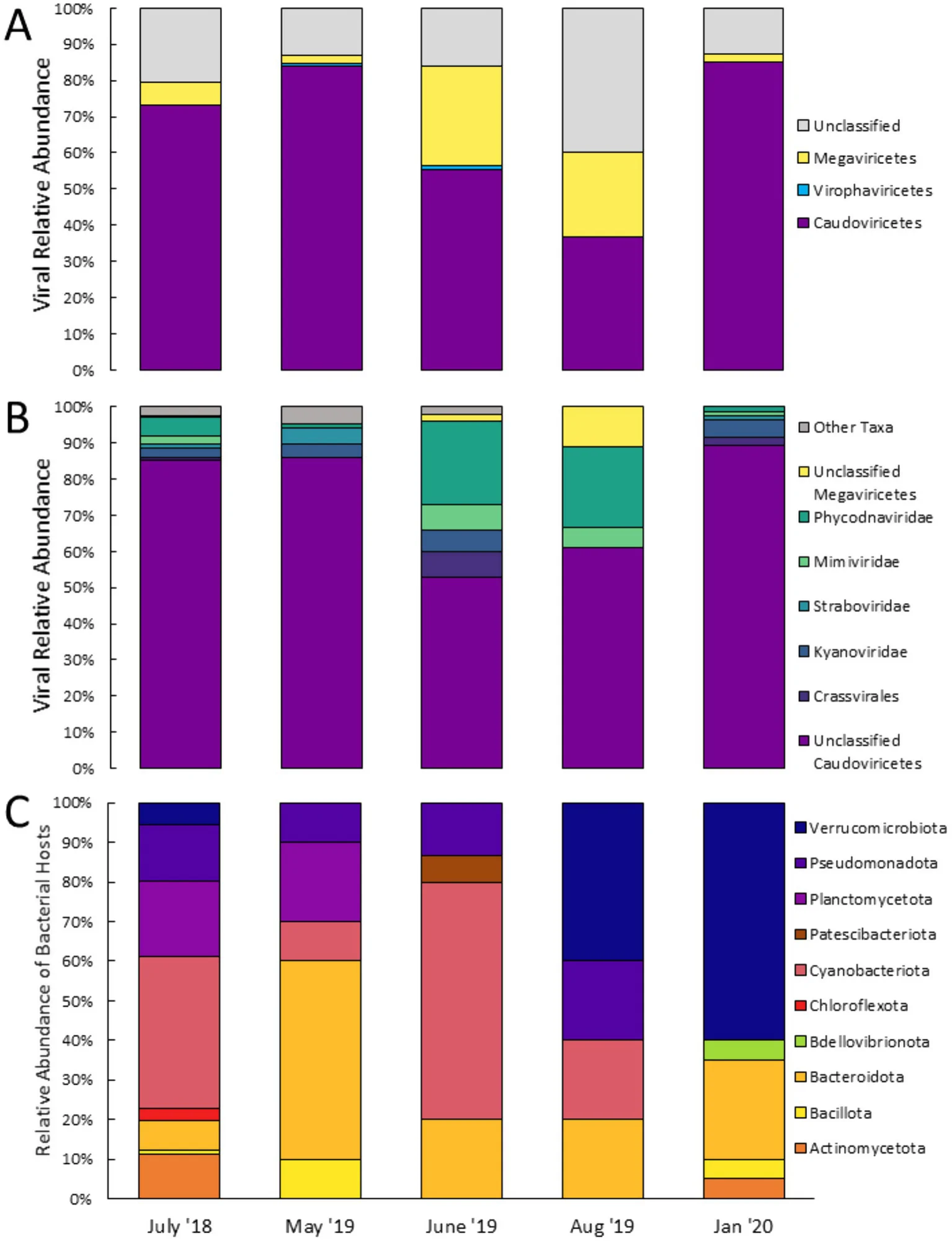
Taxonomy of cell-associated viruses and their respective hosts within Big Turkey Lake. **(A)** Relative abundance of cell-associated virus community by class. **(B)** Relative abundance of cell-associated virus community by family, omitting unclassified sequences. **(C)** Host prediction analysis using IPHoP for cell-associated classified and unclassified viruses across seasons.

Due to the abolishment of the bacteriophage families *Myoviridae, Siphoviridae*, and *Podoviridae* in 2023 (Turner et al., 2023), many bacteriophage sequences now find themselves without a corresponding viral family. This can be seen in deeper taxonomic assignments (Figure 2B), with most sequences assigned to *Caudoviricetes* unable to be resolved any further – the sample with the highest RA of deeper *Caudoviricetes* taxonomy was August 2019 with only 18% assigned family-level taxonomy. Generally, those that were able to be assigned taxonomy past *Caudoviricetes* appeared to be ephemeral; viral families *Straboviridae* and *Kyanoviridae* were only found in three and four of the total samples respectively, while the order *Crassvirales* was found only in three of the samples. In addition, these families (and order, in the case of *Crassvirales*), were not particularly abundant, with the highest RA being *Crassvirales* in June 2019 (5%). *Ackermannviridae, Autographivirales, Demerecviridae,* and *Herelleviridae* were assigned at >1% RA and were included under “other taxa” (Figure 2B). In comparison, most sequences of the class *Megaviricetes* were assigned a family-level taxonomy. The two main families were assigned as either *Phycodnaviridae* or *Mimiviridae,* viruses that infect primarily eukaryotic algae and protists, respectively. Both families were persistent across multiple samples, with higher RA in June 2019 and August 2019. Additionally, *Phycodnaviridae* and *Mimiviridae* showed a decline in RA in the winter sample of January 2020, indicating that viruses from *Megaviricetes* could have dynamic abundances that change with seasons.

One possible explanation for the differences seen between samples and across seasons may be the number of raw reads obtained after sequencing, resulting in a varied number of assembled contigs (Supplementary Table S1). This could have potentially skewed our analysis and introduced a bias that would have omitted from detection certain families or their identified RA. However, rarefaction analysis (Supplementary Figure S1) indicates that there was sufficient sequencing depth to cover the full extent of the cell-associated viral diversity in our samples. A more likely explanation is that the observed differences in RA and its diversity represent true temporal variations of the cell-associated virus community within BTL, which is indicative of its dynamic nature. Indeed, there are numerous factors that can affect the dynamic nature of a microbial community in a lake such as BTL. These include host range and the type of microbial host community, dissolved organic compound (DOC) composition, environmental conditions, dispersal mechanisms, seasonal patterns, evolutionary adaptations, infection dynamics (e.g., adsorption rates, burst sizes, and latent periods), resistance to environmental degradation, and competitive interactions between viruses over the same host for infection (Clokie et al., 2011; Mohiuddin and Schellhorn, 2015; Batinovic et al., 2019; Li et al., 2021; Sandor et al., 2026).

Finally, we also detected two virophage signatures (i.e., satellite viruses that co-infect with large algal viruses) belonging to the class *Virophaviricetes* (Figure 2A), one in May 2019, and the other in June 2019. While virophages are still a relatively underrepresented group of viruses in databases, Fischer (2021) notes that the majority of virophages recovered from metagenomes so far are from lakes. It is therefore not surprising that we detect them in our metagenomic analysis. However, it is surprising that their RA was low in our study, given prior work that has shown that up to two thirds of the virus community in a lake may consist of virophages (Palermo et al., 2019). Virophages are mostly associated with *Mimiviridae* (Tokarz-Deptuła et al., 2023), and while *Mimiviridae* are present within BTL, they do not appear to be particularly abundant in comparison to other viruses. We therefore conclude that protist viruses from *Mimiviridae* and virophages likely play a lesser role in BTL compared to in other aquatic habitats.

### 3.2 Host identification of the bacteriophage community

To gain insights about the hosts with which the Big Turkey Lake (BTL) cell-associated virus community is associated with, we ran all viral contigs (total of 799; Supplementary Table S1) through the iPHoP pipeline, which is specifically designed to computationally predict bacterial and archaeal hosts from viral data (Roux et al., 2023). This analysis was able to predict hosts (Figure 2C) for 19.5% of the viral contigs, the majority of which had an iPHoP confidence score of 90–96%.

Virus hosts belonging to the phyla *Bacteriodota* were predicted in all samples across the different seasons. This is in accordance with prior findings that report on the presence of these phyla across those samples in BTL (Cameron et al., 2022; Cameron et al., 2024). Hosts belonging to the phylum *Cyanobacteriota* were predicted for all samples apart from January 2020. This is in accordance with findings by Cameron et al. (2022) that show a near complete absence of cyanobacteria in the ice-covered months of 2020, in both BTL and the rest of the lakes that make up the TLW.

Similarly, hosts belonging to the phylum *Pseudomonadota* were predicted in all samples apart from January 2020. A lack of *Pseudomonadota* host predictions for January is surprising, given that their RA in that sample was reported by Cameron et al. (2022) to be >50% of the total bacterial community. There are two possible explanations for this discrepancy. The first is that viruses are indeed associated with *Pseudomonadota* in the ice-covered winter months in BTL, but the iPHoP pipeline failed to predict hosts for the viral contigs in this sample given the low percentage of host predictions for identified viral contigs. The second is that *Pseudomonadota* in BTL is free of viral predation in the winter, and that their viruses are within the “free fraction” of the virus community (e.g., not cell associated).

Host predictions belonging to the phylum *Verrucomicrobiota* were dominant in August 2019 and January 2020, where they comprised 40 and 60% of the total predictions in those samples, respectively; however, *Verrucomicrobiota* were not assigned as viral hosts in either May 2019 or June 2019 despite this phylum being previously detected (Cameron et al., 2022). Although we cannot rule out active infection of *Verrucomicrobiota* entirely, this may suggest real temporal variation in BTL between actively infecting viruses associated with *Verrucomicrobiota* members in some of our samples, and those that are in the “free fraction” in the rest of our samples, which would not be detected based on our methods.

Finally, our host predictions were able to also provide new insights on the genus level. Some of the viral contigs in our July 2018 sample were predicted to infect members of the *Cyanobium* (unicellular cyanobacteria; iPHoP confidence score of 91–93), *Vulcanococcus* (cyanobacteria capable of forming microcolonies; iPHoP confidence score of 90–92.5), and *Tolypothrix* (filamentous cyanobacteria’; iPHoP confidence score of 90.3) genera. To the best of our knowledge this is the first report that identifies possible virus signatures associated with sequences attributed to these cyanobacterial genera.

### 3.3 Auxiliary metabolic genes in BTL

Viral hits to candidate AMGs were found within BTL across all samples with seasonal variation in the number of AMGs found (Figure 3; Supplementary Table S3). Of the total 62 AMG candidates, 44 had one potential viral signature in a flanking region, designating them as potential AMGs, and 18 had a viral signature on both flanks, designating them as a high-confidence AMG (Supplementary Table S3). Collectively, potential AMGs + high-confidence AMGs for nucleotide metabolism were the most abundant, with a combined total of 23 genes. Within those, there were multiple genes such as guanine reductase (*guaC*) and ribonucleoside-diphosphate reductase (*nrdA*) represented across multiple samples. AMGs in this category can bolster the biosynthesis of nucleic acids, which is an important step in viral replication (Gao et al., 2016; Crummett et al., 2016). In addition, there were also multiple genes involved in folate metabolism. This includes genes such as thymidylate synthase (*thyX, thyA*), and dihydrofolate reductase (*folA*), all of which have been found in viruses previously (Luo et al., 2022; He et al., 2024). Given that viruses downregulate production of host proteins (Fabricant and Kennell, 1970; Hurwitz and U’Ren, 2016), and folate is an essential nutrient for DNA and protein synthesis (Bermingham and Derrick, 2002), viruses with those potential AMGs in their possession might be modulating host folate metabolism to boost their own replication, potentially boosting the fitness of their host, or providing an indirect benefit to other microbial community members (He et al., 2024).

**Figure 3:**
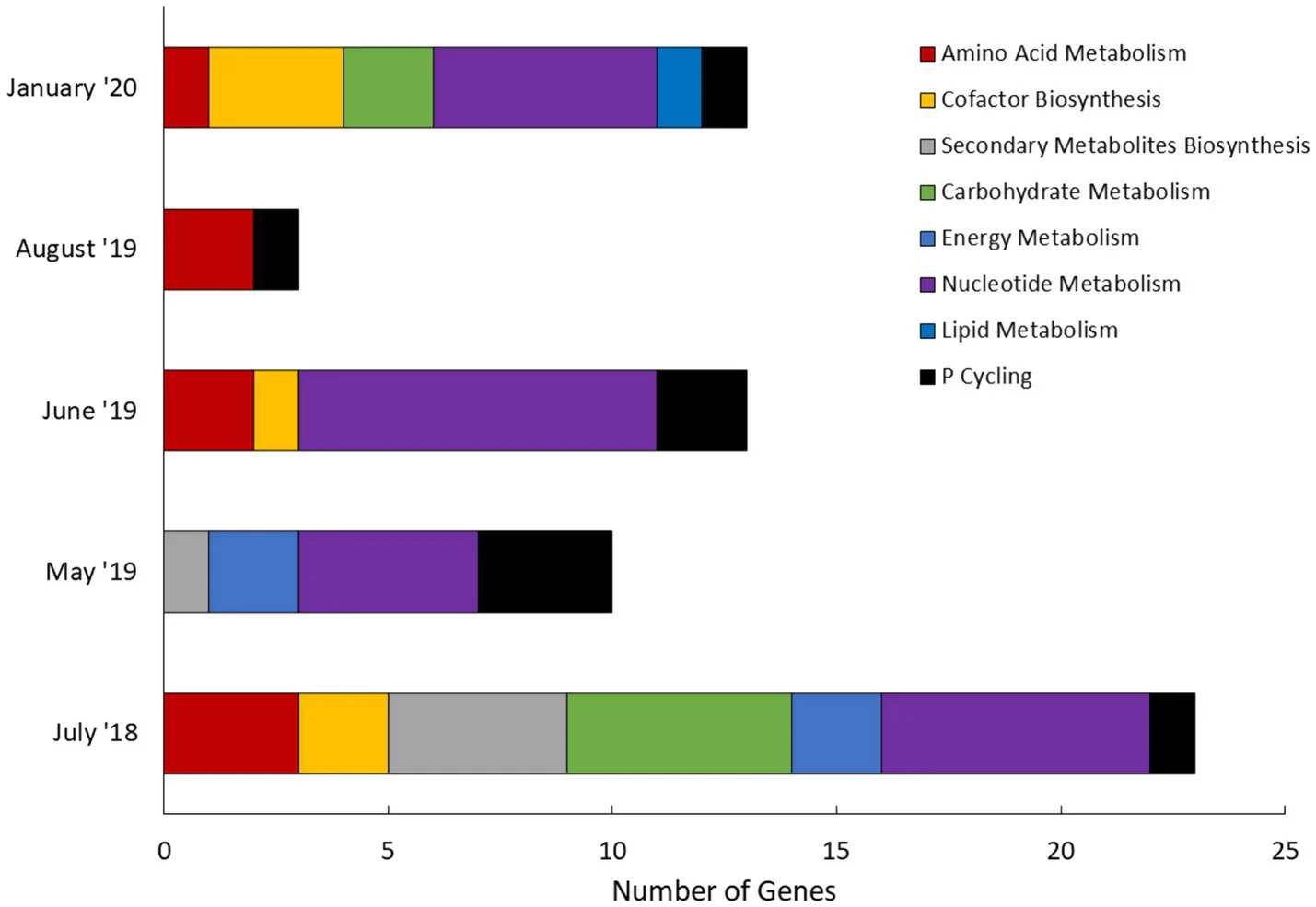
Potential and high-confidence auxiliary metabolic gene (AMG) profiles in Big Turkey Lake across samples/seasons and the number of identified genes within each AMG category.

Other key genes that we detected were those involved in energy metabolism pathways. Specifically, these are a potential AMG for succinyl-CoA synthetase alpha subunit (*sucD*), a potential AMG for photosystem II PsbK protein (*psbK*), a high-confidence AMG for nitrogen fixation protein NifU (*iscU/nifU*), and a high-confidence AMG for iron–sulfur cluster insertion protein (*erpA*); genes involved in the TCA cycle, oxygenic photosynthesis, and protein maturation (Buck et al., 1985; Ikeuchi et al., 1991; Dos Santos et al., 2004; Loiseau et al., 2007). The identification of a potential-AMG involved in photosynthesis (*psbK*) is of particular note. Oceanic phages were found to be common carriers of photosystem II genes, which makes them key players in global photosynthesis rates (Lindell et al., 2005; Heyerhoff et al., 2022). Metatranscriptomic studies show that viruses can contribute >40% of *psbA* expression within an environment (Sieradzki et al., 2019), making them key players in global oxygen production. Other AMGs identified included those involved in nitrogen fixation (*nifU*), found in May 2019; the collective presence of these AMGs in our study therefore indicates that they likely play an important role in primary productivity and nitrogen cycling in BTL.

Genes related to the phosphorus regulon and cycling (*phoH*) (Hsieh and Wanner, 2010) were found across all five samples, indicating viruses in BTL could play a role in phosphorus cycling. Given that BTL is classified as oligotrophic (Jeffries et al., 1988), and the presence of phosphorus-scavenging bacterial taxa have been previously identified (Cameron et al., 2024), the presence of genes related to phosphorus uptake have the potential to offer an evolutionary advantage for viruses that encode them. In addition, as *phoH* has been previously used as a marker for phage diversity in marine environments (Goldsmith et al., 2011), the ubiquity of *phoH* across all our samples suggests that this gene could be used as a similar diversity marker in BTL.

The identification of potential *phoH* AMGs in BTL is worthy of consideration within the broader ecological context of this watershed, as it can provide a basis for examining and integrating the viral community insights presented here within the TLW system. Changes in the composition of dissolved organic matter are increasingly identified as drivers of temporal dynamics in microbial community composition (Sandor et al., 2026). Previous work in TLW demonstrated that hydrological flow paths and event-driven connectivity strongly influence dissolved organic matter and phosphorus transport (Fines et al., 2023; Gray et al., 2024), with prior microbial community analyses repeatedly identifying the presence of phosphorus-scavenging taxa in this oligotrophic system (Cameron et al., 2024). Within this framework, the observed seasonal shifts in cell-associated viral communities and the detection of phosphorus-acquisition-related AMGs are consistent with the cell-associated (and potentially actively-infecting) viral fraction being linked to phosphorus-efficient microbial hosts. Collectively, these findings suggest likely coupling between watershed-scale transport dynamics, seasonal microbial succession, and host-viral interactions within BTL, and potentially the TLW system as a whole.

Interestingly, a high-confidence AMG involved in lipid metabolism was only detected in January 2020. The gene in question, 1,2-diacylglycerol beta-glucosyltransferase (*ugtP*), is important in the biosynthesis of membrane lipids (Matsuoka et al., 2016). Its detection in the winter is thus intriguing, given the fact that some bacteria adapt to cold temperatures by increasing production of membrane lipids and fatty acids (Hassan et al., 2020).

### 3.4 Viral metagenome-assembled genomes (vMAGs) from BTL

From our metagenomic data, we constructed 103 viral bins (representing a total of 360 viral contigs) that spanned across all five samples. Analysis of these bins by CheckV indicated that only six had a completeness of >90%, which made them candidates for vMAG consideration. These vMAG candidates had a genome size range of 11–45 kbp (Figure 4). Further analysis with the IMG/VR database revealed that all six vMAGs generated from BTL were aligned with *Caudoviricetes* phages (Supplementary Table S4). In addition, four of the six vMAGs were able to be assigned host phyla using the iPHoP pipeline, with two being assigned to *Bacteriodota*, one to *Cyanobacteriota*, and one to *Pseudomonadota*.

**Figure 4:**
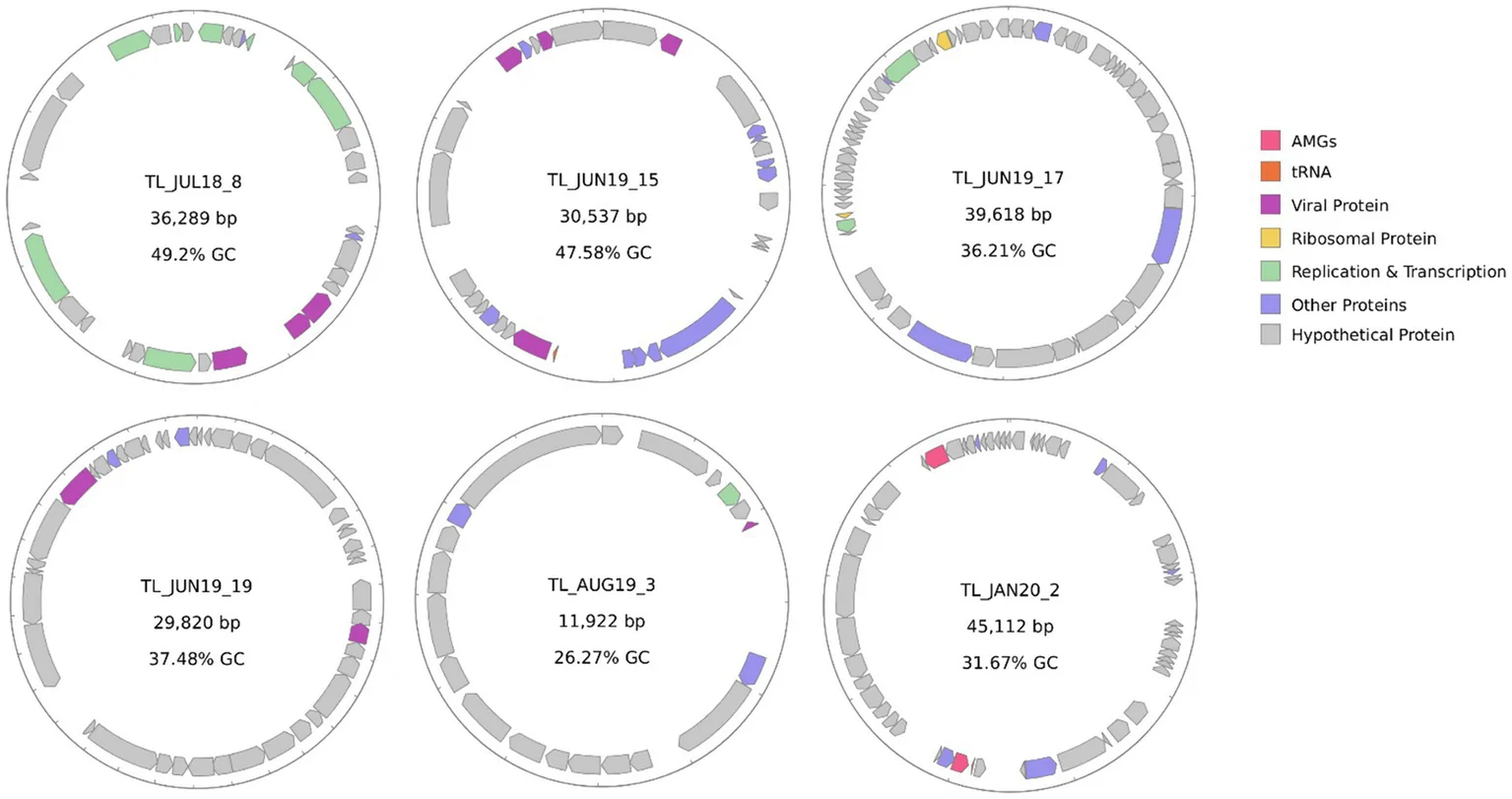
Viral Metagenome-Assembled Genomes (vMAGs) in Big Turkey Lake. vMAGs are circularized for visual clarity using *gbdraw* software. Colors represent structural viral proteins, other annotated proteins, hypothetical proteins (unknown function), tRNAs, virus proteins related to replication and transcription, AMGs, and ribosomal proteins (see Supplementary Table S5 for more details). Note that although the latter can be considered too as AMGs, they are indicated here separately to highlight the significance of their detection.

From the annotated genes (Supplementary Table S5), we found that two vMAGs (bin 8 and bin 15) contained coding regions for integrases, indicating a possible temperate lifecycle. Coding regions for tRNAs were also annotated in bin 2 and bin 15, for histidine and serine, respectively. Annotations in bin 17 included two proteins involved with ribosomes – specifically, ribosomal protein L18 and 23S rRNA/tRNA-pseudouridylate synthase. While the separation between viruses and other forms of life has been previously defined by the absence of ribosomes (Raoult and Forterre, 2008), bacteriophages have been found to encode ribosomal proteins, with the current highest number being six within one jumbophage (Meza-Padilla et al., 2026).

Currently, very few freshwater studies in Canada have reported on vMAGs (Mohiuddin and Schellhorn, 2015), leading to an under-representation of northern freshwater viruses. Assembly of novel viral genomes from uncultivated viruses is essential to increase our understanding of virus-host connections and their ecological implications (Nayfach et al., 2021a,b).

## 4 Conclusion

Our work focused only on viruses associated with cells and did not include an analysis of “free living” viruses within the water column, or of those locked within the sediment of Big Turkey Lake. Nevertheless, it provides novel insights into the virus community that is likely actively infecting microbial hosts in this lake across seasons. We reveal (i) a virus community dominated by *Caudoviricetes,* along with a large proportion of unclassified viruses, suggesting that many of the viruses in BTL are unique; (ii) that the cell-associated virus community in BTL is temporally dynamic, and (iii) that it is possible to assemble novel virus genomes even from small amounts of data, originating from a cellular microbial fraction.

We also illustrate the power of machine learning tools such as iPHoP for predicting the hosts that classified and unclassified viruses are expected to be actively infecting in a setting such as BTL. In our work, this tool enabled a glimpse into the identity of the microbial hosts that were likely being infected in different seasons, a critical aspect in improving our understanding of the role of viruses in freshwater. Importantly, this tool allowed us to report for the first time on potential cyanophage associations to *Cyanobium*, *Vulcanococcus*, and *Tolypothrix*.

Finally, we report on functionally and ecologically important viral genes such as AMGs and hypothesize on their potential role within BTL. Based on the limited data that we have, it is difficult to implicate which specific ecological, biological and physicochemical parameters might be driving their distribution or influence the AMG profile across our sampled seasons. Such profiles are driven predominantly by viral lifestyle (lytic vs. lysogenic), habitat characteristics, and host identities (Luo et al., 2022); all aspects that should be included in future investigations.

## Data Availability

The metagenomic reads from this study can be found in the NCBI SRA under the accession number SRP461269. Accession numbers for the generated vMAGs are PP191267, PP191268, PP191269, PP191270, PP191271, and PP191272.
