# Supplementary material for: Cell-associated viral community composition and its functional potential in a dimictic lake on the Canadian shield
Source: Front Microbiol. 2026 Oct 27;17:1941165. doi: 10.3389/fmicb.2026.1941165 (PMC13760526; doi:10.3389/fmicb.2026.1941165)
Supplement: Supplementary file 1 [file Supplementary_file_1.DOCX]

Supplementary Material

# 1 Supplementary Figures

**Supplementary Figure 1.** Rarefaction curves on viral community of Big Turkey Lake samples, showing Shannon diversity index at various sequencing depths.

# 2 Supplementary Tables

**Supplementary Table 1.** Sample summary from Big Turkey Lake. The samples from 2018 weas taken from 1 meter below Secchi depth, while those sampled from 2019 were taken at Secchi depth. *Due to ice cover at the time of sampling, the January 2020 sample was taken at surface level (e.g., 25 cm below the ice).

**Supplementary Table 2.** Quality control of sequncind samples from Big Turkey Lake was performed using the Metagenome-Atlas pipeline.

**Supplementary Table 3.** Auxiliary metabolic gene (AMG) candidates in Big Turkey Lake. Viral flanking regions were defined as regions surrounding the gene in question with either known viral genes, or a region of very short, hypothetical proteins.Potential AMGs are those with only one viral flanking region. AMGs are those with two. Pathways were assigned based on the KEGG Orthology database.

**Supplementary Table 4**. Big Turkey Lake viral metagenome assembled Genome (vMAG) summary and host prediction. Only vMAGs with a predicted completeness of >90% by CheckV were designated a ‘High-Quality’ vMAGs and used for further analysis.

**Supplementary Table 5.** Predicted protein annotations in the vMAGs from Big Turkey Lake as annotated by IMG/ER.
